# Evaluation of coagulation parameters in patients with parathyroid adenoma

**DOI:** 10.1038/s41598-020-76167-2

**Published:** 2020-11-05

**Authors:** Murat Alay, Berrak Mermit Ercek, Gulcin Miyase Sonmez, Aysegul Sakin, Rifki Ucler, Saliha Yildiz

**Affiliations:** 1 Department of Endocrinology and Metabolism, Faculty of Medicine, Yuzuncu Yil University, 65040 Van, Turkey; 2 Department of Internal Medicine, Faculty of Medicine, Yuzuncu Yil University, 65040 Van, Turkey; 3 Department of Internal Medicine, University of Health Sciences, Van Training and Research hospital, Van, Turkey

**Keywords:** Endocrinology, Risk factors

## Abstract

Parathyroid adenoma is responsible for 80–85% of cases of primary hyperparathyroidism. Increased fibrinogen levels in patients with adenoma may increase the risk of atherosclerosis and cardiovascular events. The aim of this study was to investigate the relationship between coagulation parameters and parathyroid adenoma. A prospective study included 28 female patients with parathyroid adenoma aged 40–88 years and 27 age-matched healthy controls. The coagulation parameters were assessed for each participant. The mean ages of the patient and control groups were 57.7 ± 10.9 and 53.3 ± 9.31 years, respectively. The mean level of protein S activity was 65.79 ± 13.78 in the patient group and 77.00 ± 15.72 in the control group, and the difference was statistically significant (*p* = 0.013). The mean fibrinogen levels of the patient and control groups were 338.78 ± 63.87 mg/dL and 304.30 ± 45.67 mg/dL, respectively, and a significant difference was found (*p* = 0.041). However, no significant difference was evident between the two groups with regard to the D-dimer (*p* = 0.238), aPTT (*p* = 0.645), INR (*p* = 0.406), protein C (*p* = 0.076), and AT-III (*p* = 0.180) levels. A positive correlation was observed between adenoma volume and fibrinogen in the patient group (*r* = 0.711, *p* = 0.001). The protein S levels were lower and the fibrinogen levels higher in the patients with parathyroid adenoma.

## Introduction

Primary hyperparathyroidism (PHPT) is a clinical condition characterized by autonomous secretions of parathyroid hormone (PTH)^1^. PHPT is the third most common endocrine disorder and the leading etiological cause of hypercalcemia in nonhospitalized patients. Although its peak incidence occurs in the fifth decade, PHPT can be seen in early ages as well. Its incidence is 2–3 times higher in women than in men^2^. Moreover, its incidence in postmenopausal women is 1:200^3^. Almost 80–85% of PHPT cases are caused by solitary parathyroid adenoma. The symptoms of PHPT associated with hypercalcemia include overt bone disease, kidney stones, and nonspecific gastrointestinal, cardiovascular, and neuromuscular dysfunction.

Hemostatic disturbances can be diagnosed by assessing the activated partial thromboplastin time (aPTT), international normalized ratio (INR), clotting time, and serum levels of protein C, protein S, and antithrombin 3 (AT-III)^4^. PHPT is typically accompanied by an increased risk of cardiovascular morbidity and mortality and has been shown to be associated with cardiovascular risk factors such as hypertension, dyslipidemia, and metabolic syndrome^5^. The coagulation abnormalities in PHPT have been reported in a limited number of case studies, and the results presented by the studies are unclear^6,7^. On the other hand, the coexistence of renal vein thrombosis and venous thrombosis-induced skin necrosis has been reported in patients with hyperparathyroidism7. PHPT has also been shown to be associated with increased factor VII (FVII), FX, D-dimer, and plasminogen activator-1 levels^8^.

The present study was designed to investigate the relationship between coagulation parameters and parathyroid adenoma.

## Results

The study included a patient group of 28 women and a control group of 27 age-matched women. Table 1 presents the demographic characteristics of both groups. No significant differences were found between the two groups with regard to age and BMI. However, both the systolic and diastolic blood pressures were significantly higher in the patient group compared to the control group (*p* = 0.004 and *p* = 0.004, respectively) (Table 1).

**Table 1:** Demographic characteristics.

Characteristics	Control group	Patient group	p*
	(n = 27)	(n = 28)	
	Mean ± SD	Mean ± SD	
BMI (Kg/m^2^)	26.41 ± 4.18	28.6 ± 5.22	0.102
Age (years) [Median (min–max)]	53.3 ± 9.31	57.7 ± 10.90	0.123
Systolic blood pressure (mm Hg)	127.10 ± 17.20	142.6 ± 20.30	0.004
Diastolic blood pressure (mm Hg)	78.10 ± 4.91	83.40 ± 7.30	0.004

*BMI* Body mass index; *SD* standard deviation.

*Independent samples t-test.

The serum P (*p* = 0.001) and albumin (*p* = 0.018) levels were significantly lower in the patient group compared to the control group. In contrast, the PTH (*p* = 0.000) and Ca (*p* = 0.000) levels were significantly higher in the patient group compared to the control group. Table 2 presents the laboratory parameters of both groups (Table 2).

**Table 2:** Laboratory parameters between study groups.

Characteristics	Control group	Patient group	p*
	(n = 27)	(n = 28)	
	Mean ± SD	Mean ± SD	
Ca (mg/dl)	9.62 ± 0.66	10.75 ± 0.67	< 0.001
P (mg/dl)	3.24 ± 0.60	2.53 ± 0.83	0.001
PTH (Pg/ml)	76.18 ± 31.02	203.91 ± 15.87	< 0.001
Albumin (g/dl)	4.59 ± 0.37	4.33 ± 0.41	0.018
UCa/Cr in spot urine samples	0.13 ± 0.10	0.19 ± 0.16	0.140
aPTT	30.96 ± 4.23	32.00 ± 5.43	0.645
Fibrinogen (mg/dl)	304.30 ± 45.67	338.78 ± 63.87	0.041
D-dimer (μg/L)	1.31 ± 0.85	1.03 ± 0.85	0.238
INR	1.12 ± 0.21	1.29 ± 0.15	0.406
Protein C (%)	81.35 ± 6.20	85.50 ± 7.75	0.076
Protein S (%)	77.00 ± 15.72	65.79 ± 13.78	0.013
AT-III (%)	75.73 ± 12.66	70.85 ± 13.59	0.180

*Ca* calcium; *P* phosphor; *PTH* parathyroid hormone; *aPTT* activated partial thromboplastin time; *INR* international normalized ratio; *AT-III* ant thrombin 3.

*Independent samples t-test.

In the patient group, the protein S levels were significantly lower (*p* = 0.013) and the fibrinogen levels significantly higher (*p* = 0.041) compared to the control group. However, no significant difference was found between the two groups with regard to D-dimer (*p* = 0.238), aPTT (*p* = 0.645), INR (*p* = 0.406), protein C (*p* = 0.076), and AT-III (*p* = 0.180) levels (Table 2).

**Figure 1:**
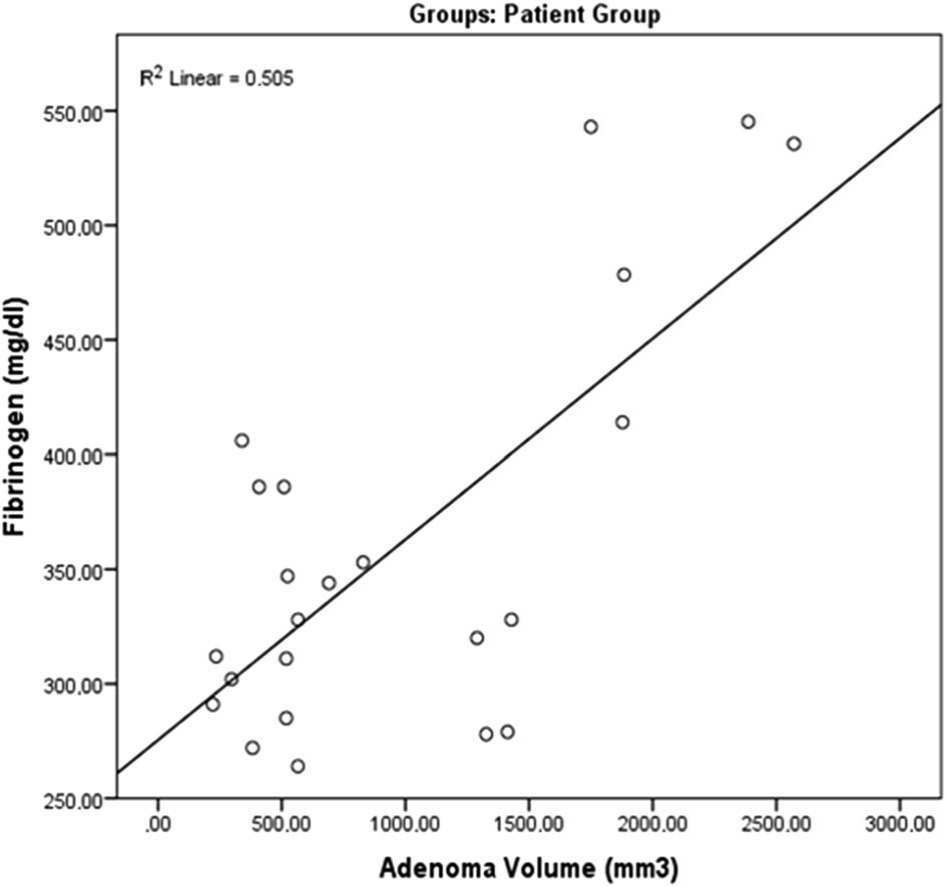
The correlation between adenoma value and fibrinogen levels.

In the patient group, a bivariate analysis indicated a significantly positive correlation between adenoma volume and fibrinogen (*r* = 0.711, *p* = 0.001). Table 3 and Fig. 1 present the correlations determined in the patient group.

**Table 3:** The correlation between adenoma value and fibrinogen levels.

	Fibrinogen (mg/dl)	PTH	Ca (mg/dl)	PR-S
**Adenoma volume (mm**^**3**^**)**				
Pearson Correlation	0.711**	0.189	0.146	0.156
Sig. (2-tailed)	< 0.001	0.411	0.477	0.445
N	28	28	28	28

*PR-S* protein S; *Ca* calcium; *PTH* parathyroid hormone.

## Discussion

Increased levels of fibrinogen in patients with parathyroid adenoma may pave the way for clotting and increase the risk of atherosclerotic and atherothrombotic complications since fibrinogen is an independent risk factor for cardiovascular disease. Accordingly, the present study aimed to investigate the relationship between coagulation parameters and hyperparathyroidism in patients diagnosed with parathyroid adenoma. The results indicated that the fibrinogen levels were significantly higher and the protein S levels significantly lower in the patient group compared to the control group. Additionally, a bivariate analysis showed a significantly positive correlation between adenoma volume and fibrinogen in the patient group.

Elbers et al. evaluated 54 patients with hyperparathyroidism secondary to vitamin D deficiency and found no significant difference between the patients and control subjects with regard to PT, aPTT, fibrinogen, FVII, and FVIII levels^9^. Yorulmaz et al. evaluated 25 patients with PHPT, 25 patients with secondary hyperthyroidism, and 25 healthy controls and revealed that the aPTT and D-dimer levels were significantly increased in the patients with secondary hyperthyroidism^10^. Taken together, these findings seem highly controversial. In our study, no significant difference was found between the patient and control groups with regard to coagulation parameters, including D-dimer, aPTT, INR, protein C, and AT-III levels.

Increased fibrinogen levels are a major risk factor for cardiovascular disease. A prospective study conducted by the Northwick Park Heart Study in the United Kingdom evaluated a total of 1,511 male patients aged 40–64 years over a period of 10 years and reported that cardiovascular events were observed in 106 patients. The study also compared the groups with a mean fibrinogen level of 290 ± 59 mg/dL and those with a mean fibrinogen level of 315 ± 71 mg/dL and found a significant relationship between a high fibrinogen level and cardiovascular risk within the first 5-year follow-up period^11^. The Atherosclerosis Risk in Communities study evaluated a total of 6,297 men and 8,180 women aged 45–64 years and reported that the mean fibrinogen levels were 295 ± 65 mg/dL and 320 ± 65 mg/dL in the men and 306 ± 65 mg/dL and 346 ± 65 mg/dL in the women over a mean follow-up period of 5.2 and 5.5 years, respectively. By comparing these values, the authors concluded that an increased fibrinogen level is a risk factor for cardiovascular disease^12^.

In the Scottish Heart Health Study 1 (SHHS 1), SHHS 2, Fowkes, and MONICA studies conducted in Scotland, the relationship between fibrinogen and cardiovascular disease was investigated. The SHHS 1 examined 3,930 men and 3,760 women aged 40–59 years without cardiovascular disease, while the SHHS 2 examined 1,163 men and 1,102 women of the same age range with cardiovascular disease. At the end of the 8-year follow-up period, the men and women in the SHHS 1 had mean fibrinogen levels of 276 md/dL and 287 mg/dL as opposed to 290 mg/dL and 311 mg/dL in the SHHS 2, respectively. Based on these values, the authors concluded that fibrinogen is an independent risk factor for cardiovascular disease^13^. Likewise, a study conducted by European Concerted Action on Thrombosis and Disabilities and, separately, the Bezafibrate Infarction Prevention study obtained similar findings and also emphasized that fibrinogen is a risk factor for cardiovascular disease^14,15^. The Prospective Cardiovascular Munster study and Gottingen Risk Incidence Prevalence Study in Germany similarly concluded that fibrinogen is an independent risk factor for cardiovascular disease^16,17^. Moreover, numerous other studies have suggested that there is a significant relationship between increased plasma fibrinogen levels and cardiovascular events, including ischemic heart disease, peripheral artery disease, stroke, and deep vein thrombosis^13^.

To our knowledge, no study in the literature has investigated the fibrinogen levels in patients with parathyroid adenoma. In our study, the fibrinogen levels were significantly higher in the patient group compared to the control group. Given that fibrinogen is an independent risk factor for cardiovascular disease, we suggest that clinicians keep atherosclerotic heart disease and cardiovascular complications in mind when treating in patients with parathyroid adenoma.

In our study, the Ca and PTH levels and systolic blood pressure were found to be significantly higher in the patient group. A previous study evaluated the association between blood pressure and the incidence of cardiovascular disease using the Framingham Risk Score and suggested that individuals with a systolic pressure of 130–139 mm Hg or a diastolic pressure of 85–89 mm Hg are at twice the risk of cardiovascular death compared to individuals with a systolic pressure of less than 120 mm Hg and diastolic pressure of less than 80 mm Hg^18^.

Patients with a protein S deficiency are prone to recurrent episodes of venous thromboembolism (VTE). However, although numerous cases of arterial thromboembolism with protein S deficiency have been reported in the literature, the clinical outcomes associated with arterial thromboembolism remain controversial. Douay et al. evaluated the protein S levels of 24 young adults with ischemic stroke and found no significant difference between the patients and control subjects^19^. In contrast, a Mexican study conducted by Martinez et al. evaluated the protein S, protein C, and AT-IIIle vels in 60 patients with ischemic stroke and reported that ischemic stroke was associated with deficiencies of these three factors^20^.

Hereditary thrombophilias are considered to play a key role in pulmonary embolism and its recurrence. Turan et al. evaluated 90 patients diagnosed with pulmonary embolism by conducting thrombophilia screening, which included screening for mutations of Factor V Leiden, prothrombin G20210A, methylenetetrahydrofolate reductase C677T-A1298C, and antithrombin III and serum levels of protein C, protein S, factor VIII, and activated protein C resistance. The authors found a significant relationship between recurrent pulmonary embolism (10 patients, 12.2%) and protein S deficiency^21^. Additionally, other studies have indicated that protein C and S deficiencies increase the risk of thromboembolism by 2–11 times when compared to the general population^20,21^. On the other hand, Onderoglu et al. evaluated factors affecting hereditary thrombophilia and reported that the incidence of protein C and S deficiencies was significantly higher in their patient group compared to the control group^22^.

To our knowledge, no study in the literature has examined the protein S levels in patients with parathyroid adenoma. The present study is therefore the first to show that the protein S levels were significantly lower in the patient group compared to the control group.

The measurement of protein S is quite complex as it can be found both free and bound in plasma.

Notwithstanding, protein S levels are likely to change, even within the same day, as a result of various factors such as age, gender, race/ethnicity, and pregnancy. In fact, a diagnosis of protein S deficiency could be missed if antigen levels alone are measured, and a quantitative deficiency of protein S could be missed if protein S activity alone is measured. Moreover, the assessment of protein S activity is likely to produce false-negative results. For these reasons, the combined use of functional (protein S activity) and immunologic (free and total protein S antigen) laboratory tests could be more beneficial for the diagnosis of protein S deficiency^23^.

To the best of our knowledge, no study in the literature has examined coagulation parameters in patients with parathyroid adenoma. The strengths of our study are that it is the first study on this subject and that it was carried out prospectively. A limitation of our study was the small sample size due to the rarity of parathyroid adenomas. The combined use of functional (protein S activity) and immunologic (free and total protein S antigen) laboratory tests could be more beneficial for the diagnosis of protein S deficiency. A further limitation of our study was therefore that these tests were not performed together to diagnose protein S deficiency.

In conclusion, we found significant differences between the patients with parathyroid adenoma and the healthy controls with regard to serum levels of coagulation parameters. Based on these differences, we propose that the increased fibrinogen activity in patients with parathyroid adenoma may pave the way for clotting and that fibrinogen increases the risk of atherosclerotic and atherothrombotic complications in these patients since it is an independent risk factor for cardiovascular disease and leads to increased systolic blood pressure. On the other hand, the lower protein S levels detected in our patients compared to those of the healthy controls could be an indication of an increased tendency for excessive blood clotting and may also increase the risk of VTE and ischemic stroke in patients with parathyroid adenoma. Despite the progress in primary and secondary prevention, cardiovascular disease remains one of the leading causes of mortality and morbidity.

Our results may serve to guide clinicians in the monitoring of complications related to thrombosis in patients with parathyroid adenoma. Further studies with a larger patient series are needed to substantiate our findings.

## Materials and methods

### Study design and patients

This prospective study included 30 female patients aged 19–88 years who were followed up in our endocrine diseases outpatient clinic due to parathyroid adenoma between February 2018 and December 2019. Pregnancy, oral contraceptive use, anticoagulant use, and a history of acute thrombosis were defined as the exclusion criteria since they could have affected the coagulation parameters. Parathyroid adenoma was diagnosed based on serum calcium, phosphor, PTH, and albumin levels as well as parathyroid ultrasound (USG) examination and the urinary calcium/creatinine ratio (UCa/Cr) in spot urine samples.

A control group that included 30 age-matched healthy women who presented to our endocrine diseases outpatient clinic and had no inherited or genetic diseases was also included in the study.

This study was performed in accordance with Good Clinical Practice and the Declaration of Helsinki. The University of Yüzüncü Yıl Investigational Review Board assessed the ethics of our procedures and approved our study (Project code: TTU-2019–8240, Date: September 11, 2019). All methods were carried out in accordance with the relevant directives and regulations, and informed consent was obtained from all the participants for the experiments.

### Measurement of routine biochemical parameters

The demographic characteristics, including age and body mass index (BMI), were recorded for each participant. Blood and urine samples were collected for the measurement of PTH, calcium (Ca), phosphor (P), albumin, and UCa/Cr in spot urine samples. The measurements were performed using an Architect Ci16200 autoanalyzer (Abbott Laboratories, Abbott Park, IL). For the participants with an albumin level of less than 4 mg/dL, the total serum Ca was corrected for albumin using the following formula: corrected Ca = total Ca + (0.8 × [4.0 − albumin]). The UCa/Cr in the spot urine samples was calculated for each participant.

Venous blood samples were collected from each participant to measure the serum aPTT, D-dimer, fibrinogen, INR, protein C, protein S, and AT-III activity. The samples were placed in coagulation tubes containing a buffered 3.2% trisodium citrate solution and then promptly transferred to our Hematology Laboratory to assess the aPTT, D-dimer, INR, and fibrinogen activity. The measurements were performed using a STA Compact Ci16200 autoanalyzer (Diagnostica Stago, Inc., Parsippany, NJ) with commercially available kits, and the normal reference range of fibrinogen activity was accepted as 180–400 mg/dL and the normal reference range of D-dimer as 0–500 μg/L.

For the assessment of protein C, protein S, and AT-III activity, the blood samples were centrifuged at 3,000 rpm/min for 10 min, and the serum was then separated. The serum samples were stored at − 20 °C until analysis (within 1–2 weeks) and analyzed using a STA Compact Ci16200 autoanalyzer with commercially available kits.

In the tests performed for the evaluation of AT-III, protein-S, and protein-C activity, the normal activity ranges were accepted as 75–125%, 60–123%, and 70–140%, respectively.

### Parathyroid USG

A neck USG examination was performed for each patient in the patient group using the GE Logiq 7 USG system (GE Healthcare, Milwaukee, WI). The localization, volume (width × length × height × π/6), and the largest diameter of the adenomas detected on USG were recorded.

### Statistical analysis

In our study, the planned sample size was at least 28 subjects, and the power analysis (power of a test) was calculated based on this sample. According to the results obtained for 28 subjects using the G*Power statistical program version 3.1.9.4 (Faul and Erdfelder, 1998), the power was calculated as 90%, the type 1 error was 5%, and the effect size was 0.8.

The data were analyzed using SPSS for Windows version 20.0 (IBM, Chicago, IL, USA). The continuous variables were expressed as mean, standard deviation, minimum, and maximum. The categorical variables were expressed as frequencies (n) and percentages (%). To determine whether the measurements in the study were normally distributed, the Shapiro–Wilk test (n < 50) was used, and it was observed that the measurements were normally distributed. The continuous variables were compared using a Student’s *t*-test, and the correlations between the continuous variables were determined using Pearson’s correlation coefficient. A *p*-value of < 0.05 was considered significant.

### Institutional review board statement

This study and all the relevant procedures were performed in accordance with the Declaration of Helsinki after obtaining ethical board approval from the Yüzüncü Yıl University Ethics Committee (Project code: TTU-2019-8240, Date: September 11, 2019).
